# Time-Resolved Fluorescence Spectroscopy and Fluorescence Lifetime Imaging Microscopy for Characterization of Dendritic Polymer Nanoparticles and Applications in Nanomedicine

**DOI:** 10.3390/molecules22010017

**Published:** 2016-12-24

**Authors:** Alexander Boreham, Robert Brodwolf, Karolina Walker, Rainer Haag, Ulrike Alexiev

**Affiliations:** 1 Institut für Experimentalphysik, Freie Universität Berlin, Arnimallee 14, 14195 Berlin, Germany; alexander.boreham@fu-berlin.de (A.B.); brodwolf@zedat.fu-berlin.de (R.B.); 2 Institut für Chemie und Biochemie, Freie Universität Berlin, Takustrasse 3, 14195 Berlin, Germany; karolina.walker@fu-berlin.de; 3 Helmholtz Virtual Institute—Multifunctional Biomaterials for Medicine, Helmholtz-Zentrum Geesthacht, Kantstr. 55, 14513 Teltow, Germany

**Keywords:** nanomedicine, dendritic nanoparticles, time-resolved fluorescence depolarization, fluorescence lifetime imaging microscopy, FLIM

## Abstract

The emerging field of nanomedicine provides new approaches for the diagnosis and treatment of diseases, for symptom relief and for monitoring of disease progression. One route of realizing this approach is through carefully constructed nanoparticles. Due to the small size inherent to the nanoparticles a proper characterization is not trivial. This review highlights the application of time-resolved fluorescence spectroscopy and fluorescence lifetime imaging microscopy (FLIM) for the analysis of nanoparticles, covering aspects ranging from molecular properties to particle detection in tissue samples. The latter technique is particularly important as FLIM allows for distinguishing of target molecules from the autofluorescent background and, due to the environmental sensitivity of the fluorescence lifetime, also offers insights into the local environment of the nanoparticle or its interactions with other biomolecules. Thus, these techniques offer highly suitable tools in the fields of particle development, such as organic chemistry, and in the fields of particle application, such as in experimental dermatology or pharmaceutical research.

## 1. Introduction

### 1.1. Time-Resolved Fluorescence Spectroscopy and Imaging in Relation to Nanomedicine and Pharmaceutics

The field of nanomedicine encompasses the application of nanomaterials and devices as therapeutic or diagnostic tools in medicine with the aim of improving the diagnosis, prevention and treatment of diseases for a better understanding of the complex underlying pathophysiology of diseases, and for improving the quality of life of patients [1]. Nanoparticles are often defined as those having a size smaller than 100 nm. As a consequence of the availability of many different building blocks and the almost limitless possibilities in manipulating particle size, shape, and surface chemistry a wide range of nanoparticles has been developed [2]. However, small changes to the physicochemical properties of a nanoparticle can have a significant impact on their bioreactivity and therefore particles will need to be assessed on a case-by-case basis [2]. To fully exploit the potential of nanoparticles for nanomedical applications a clear understanding of both physicochemical and physiological processes is mandatory as these provide the underlying basis for the way nanoparticles interact with their microenvironment [3]. 

Time-resolved fluorescence spectroscopy and imaging have been successfully used in elucidating structure and function of biological samples, e.g., proteins [4], and also have the potential to provide an extremely powerful set of tools in the evaluation of dye-conjugated polymeric materials, nanoparticles, and nanocapsules [5,6]. However, in the study of nanoparticles time-resolved fluorescence measurements have not been used as much as steady-state approaches, such as the analysis of fluorescence emission spectra and steady-state anisotropy, even though they offer the possibility for a deeper understanding of the nanoparticle itself and the local environment as well as being able to provide information on loaded drug molecules.

Time-resolved recordings of the fluorescence emission, i.e., the fluorescence lifetime curves, are highly sensitive to a fluorophores environment and the respective fluorescence decay curves can thus be used to gain insights into dye-nanoparticle conjugate systems, such as the validation of conjugation, the interaction of nanoparticles with their environment, but also into the distribution and environment of guest molecules within nanoparticles. Furthermore, time-resolved measurements of the fluorescence depolarization, i.e., the fluorescence anisotropy, can be used to determine the size of nanoparticles and in addition they also contain information on the conformational flexibility of the nanoparticle structure and on the conformational space of loaded guest molecules. Recording the time-resolved fluorescence emission in a spatially resolved fashion on samples under a microscope, i.e., fluorescence lifetime imaging microscopy (FLIM), can provide localized information on fluorophores in cell and tissue samples. The use of FLIM includes environmental sensing of, amongst others, polarity, local pH, oxygen and calcium concentrations, as well as the study of biomolecular interactions in living cells (e.g., [7,8,9]). There is a large variety of different fluorescent probes available, also in nanoparticulate form, that are widely used in fluorescent imaging (for a review see [10]). New developments in the analysis of fluorescence lifetime imaging microscopy data enable fast and reliable localization of target molecules, e.g., dye-nanoparticle conjugates, despite an autofluorescent background, thus allowing for the detection of fluorescently labeled nanoparticles in cellular systems and tissue samples [11,12,13,14,15,16]. 

### 1.2. Dendritic Nanoparticles

In the field of nanomedicine the vast diversity of nanoparticles ranges from hard inorganic and metallic nanoparticles to soft nature-derived or synthetic polymer-based nanoparticles [1,3,6,17,18,19]. Within this spectrum one can find dendritic polymer-based nanoparticles, in the following termed as dendritic nanoparticles. This review will highlight how time-resolved fluorescence spectroscopy and imaging can be used to gain insights into diverse molecular properties of dendritic nanoparticles and show how these techniques were used to investigate the mode of action of dendritic nanoparticles.

For a better understanding, the nanoparticle systems serving as model systems in the context of this review will be introduced in the following. Among polymeric nanoparticles there are two major classes: plain nanoparticles made from aggregating individual polymeric molecules and dendritic nanoparticles, which are based on a branched focal point surrounded by covalently-attached molecules. Nanoparticles which are based on the aggregation of individual polymeric molecules are only stable at concentrations above the critical aggregation concentration, and are unstable in high dilution, i.e., they disassemble at low concentrations. In contrast to that, dendritic nanoparticles are unimolecular nanoparticles, and they are stable even under high dilution [20]. Further benefits of dendritic over plain nanoparticles are their good solubility in different solvents, their well-defined structure, and the large number of functional groups at the surface which can be used for further modifications. The combination of these features makes dendritic molecules interesting for a broad spectrum of applications, e.g., as multifunctional polymeric support in catalysis, as a template for the synthesis of inorganic nanoparticles, as environmentally sensitive dendritic fluorescent or phosphorescent probes, amongst others for oxygen imaging [21], and even for energy harvesting and storage [22]. Nevertheless, the dominant field of application of dendritic molecules is the delivery of agents, namely the delivery of drugs, dyes, contrast agents, and even genes [20,23]. As the synthesis of dendritic molecules is often based on a modular approach, the needs for different applications can be met by the introduction of tailor-made building blocks. This allows for example the rational design of nanoparticles for the delivery of specific drugs, and an optimal solubility and mode of action in the intended medium. The so-called core building block with its dendritic, i.e., tree-like architecture offers voids in their interior, which are exploited as cavities for the entrapment of guest molecules. These features enable dendritic nanoparticles for both the use in therapeutic as well as diagnostic applications [24,25]. 

**Figure 1:**
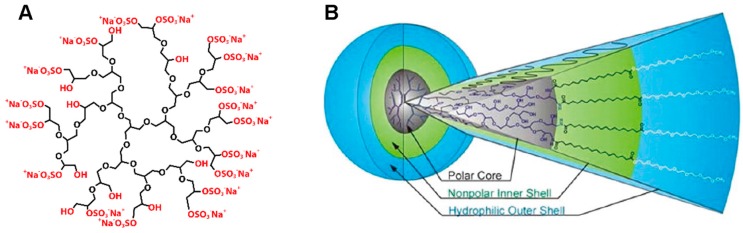
Dendritic polyglycerol sulfate and core-multishell nanocarrier architecture. (**A**) Schematic representation of a highly anti-inflammatory dendritic PG sulfate (dPGS); (**B**) Schematic representation of the dendritic core multishell (CMS) architecture (A: reprinted from Calderón et al., [28] by permission from John Wiley and Sons © 2010; B: reprinted from Fleige et al. [34] with permission of The American Chemical Society © 2012).

A representative of dendritic nanoparticles is dendritic polyglycerol sulfate (dPGS). This molecule is characterized by a highly ordered, branched architecture, a globular shape and negatively charged sulfate groups at the surface, leading to an overall biocompatible nanoparticle (Figure 1A) [26,27,28]. dPGS has been demonstrated to be bioactive [26] and has been used as a biomarker [25,29]. The anti-inflammatory activity has been shown to be mediated by the binding of dPGS to selectins [26,30]. This behavior was found to be specific for dPGS and was not found for other polyanions [31]. A recent study identified a complex structure-activity relationship, implying that multivalency, particle size, as well as the structural flexibility all define and modulate the biological activity of dPGS [32,33].

dPGS is based on dendritic polyglycerol (dPG), depicted in black in Figure 1A. Instead of modifying the surface of the nanoparticle with sulfate groups as in dPGS, dPG can as well be used as a branching scaffold in the synthesis of the so-called core multishell (CMS) nanocarrier. In that case amphiphilic water-soluble linear molecules serving as a double shell are attached to the large amount of functional groups of dPG, leading to a liposome-like particle with different polarities of the segments (Figure 1B): the polar dPG core, surrounded by a nonpolar alkyl chain as the inner shell and a hydrophilic methyl ether PEG (mPEG) as the outer shell (Figure 1B) [35,36]. These nanoparticles have been shown to be good carriers for hydrophobic, poorly water-soluble drugs [35,37]. Furthermore, CMS nanocarriers have been shown to target tumor tissue [27] and have been studied for the delivery of anti-inflammatory drugs and pain killers [38]. Recently published studies demonstrate the biocompatibility of these CMS nanocarriers, tested on in vitro and in vivo skin disease models [39,40].

The transport of drugs can be accomplished by either covalent attachment of the drug to the carrier or by the physical entrapment of the drug inside the carrier or carrier aggregates, generally referred to as encapsulation [34]. The field of dendritic nanoparticles has developed a large span of particles differing in their intrinsic material properties and particle sizes. Based on the library of various materials and building blocks, tailor made particles with features like targeting moieties, on-demand release or cleavage of a drug, and degradation of the particle can be realized [20,41]. As an example, pH-responsive dendritic core-multishell nanocarriers have been reported, allowing for cleavage of the CMS shells from the core at acidic pH thereby leading to more efficient drug release [11]. 

In this review we highlight the use of time-resolved fluorescence spectroscopy in “cuvette” experiments to characterize the molecular properties of dendritic nanoparticles, such as size, and conformational dynamics of the polymer branches, as well as cargo partitioning and biomolecular interactions by using a classical organic fluorophore as a reporter group. Furthermore, we provide insight on how the sensitivity of the fluorescence lifetime to the microenvironment can be used in FLIM experiments to localize these nanoparticles in cells and tissue sections. This review is primarily intended for pharmaceutical and medical researchers.

## 2. Basic Concepts of Time-Resolved Fluorescence

Fluorescence occurs upon relaxation of an excited-state electron to the ground state by photon emission. Molecules with the ability to fluoresce are known as fluorescent dyes, fluorophores or, as often found in biomedical literature, fluorochromes. Fluorescence spectroscopy and microscopy have become very useful and powerful tools, not only in modern day biophysics, but also for high throughput screening in pharmaceutical research [42,43,44,45]. From steady-state to time-resolved fluorescence, from fluorescence emission spectra to state-of-the-art single-molecule methods, fluorescence measurements have been extensively used to study and visualize localization, interaction and dynamics of biomolecules (e.g., [4]). In the following, the main aspects of fluorescence relevant for the application of time-resolved fluorescence spectroscopy and imaging of nanoparticles in nanomedicine will be discussed. This includes the fluorescence lifetime, an environmental sensitive parameter which can, for example, report on the distribution of loaded drug molecules, fluorescence quenching, which can be used to determine molecular interactions, and the fluorescence time-resolved fluorescence anisotropy, containing information on successful drug binding or incorporation, on nanoparticle size and conformational flexibility.

### 2.1. Fluorescence Properties and Lifetime

**Figure 2:**
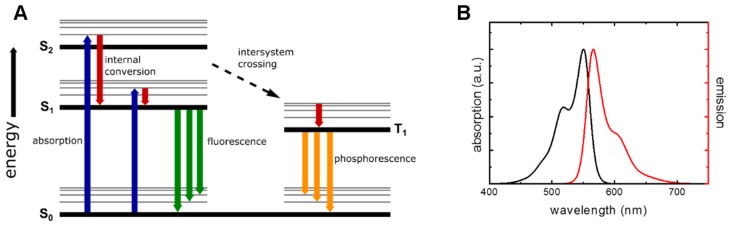
(**A**) Jablonski diagram depicting energy transitions between the possible electronic states of a fluorophore: photon absorption in blue, photon emission in green, vibrational relaxation, internal conversion in red, intersystem crossing as a dashed black line and phosphorescence in yellow. Singlet states are abbreviated as S and triplet states as T; (**B**) Absorption (black) and emission (red) spectra of an indocarbocyanine (ICC) dye.

The main principle of fluorescence emission is often visualized by a Jablonski diagram (Figure 2A). Absorption of a photon transfers a ground-state electron into the excited state. From here the electron will return back into the electronic ground state, either by non-radiative processes or by sending out a photon. The latter is the fluorescence emission. The fluorescence emission spectrum of a fluorophore characterizes the wavelength dependent emission of a fluorophore at a certain excitation wavelength. A characteristic of the emission spectrum is the red-shift, i.e., a shift to lower wavelengths, of the spectrum compared to the absorption spectrum, known as the Stokes-shift (Figure 2B).

Different fluorophores have different efficiencies for converting absorbed photons into fluorescence emission. This efficiency is defined by the quantum yield *η*:

(1) $$\eta =\frac{number\;of\;emitted\;photons}{number\;of\;absorbed\;photons}$$

The quantum yield is only equal to 1 if every absorbed photon is also emitted. However, this is hardly ever reached, as other ways to dissipate the energy gained by photon absorption exist. For example, the quantum yield of fluorescein is 0.79 (in aqueous solution) [46], and the quantum yield of indocarbocyanine is 0.07 (in methanol) [47].

A further characteristic property of a fluorophore is the fluorescence lifetime. The fluorescence lifetime *τ* describes the average time an exited-state electron remains in the exited state before relaxing to the ground state via photon emission. The fluorescence lifetime is dependent on the rate of photon emission $k_{r}$ and the rate of the non-radiative decay processes $k_{\mathrm{nr}}$ (Equation (2)):

(2) $$\tau =\frac{1}{k_{r}-k_{\mathrm{nr}}}$$

**Figure 3:**
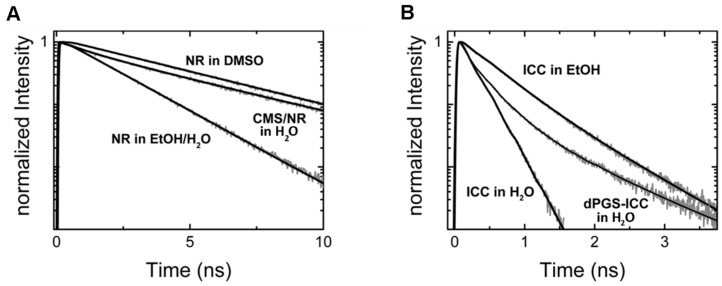
Environmental sensitivity of the fluorescence decay (fluorescence lifetime curve). (**A**) Fluorescence decay traces of the fluorescent dye Nile Red (NR) in different environments: ethanol/water (1:1) mixture, DMSO, and encapsulated within CMS nanocarriers dissolved in aqueous solution; (**B**) Fluorescence decay traces of an indocarbocyanine dye (ICC) in different environments: aqueous solution, ethanol, and covalently attached to the surface of dPGS nanoparticles (dissolved in aqueous solution).

The influence of the non-radiative decay rates is also the root of the environmental sensitivity of the fluorescence lifetime (Figure 3). While the intrinsic radiative decay rate remains unchanged, the contribution of non-radiative decay processes is strongly dependent on the fluorophores environment.

### 2.2. Fluorescence Quenching and Energy Transfer Mechanisms

Quenching describes the phenomenon of a reduced quantum yield or lifetime of a fluorophore through interactions with another molecule. Such molecules are called quenchers. Two types of quenching can be distinguished: static and dynamic quenching [48,49]. Dynamic quenching is caused by interactions of the quencher and the fluorophore in the excited state. As the interaction with a quencher represent a further way of non-radiative return into the ground state the fluorescence decay is affected. In contrast, static quenching involves the formation of non-fluorescent complexes between ground-state fluorophores and the quencher and does not affect the fluorescence decay. 

Other possible energy transfer mechanisms depopulating the excited state can be classified into short and long range mechanisms. Short range energy transfer involves the transfer of an electron between a fluorophore and a quencher. This can occur when the two molecules come into close contact and is only effective within distances up to ~1 nm. Several different mechanisms of electron transfer induced quenching are known, including Dexter mechanism [50] and photo-induced electron transfer [51]. Long range energy transfer is mediated by dipole-dipole interactions between two molecules designated as the donor (D) and the acceptor (A) molecule. This long range energy transfer mechanism is known as Förster resonance energy transfer (FRET) [52].

### 2.3. Time-Resolved Fluorescence Anisotropy

Brownian motion is the irregular displacement of particles in a gas or liquid resulting from collisions, induced by thermal agitation, between the particles themselves and the surrounding atoms or molecules. It leads to both lateral and rotational diffusion of the particles. The rotational diffusion is dependent on the rotating particle’s size and shape. Assuming a spherical particle the rotational diffusion coefficient $D_{\mathrm{rot}}$ can be expressed as:

(3) $$D_{\mathrm{rot}}=\frac{k_{B}T}{8\pi \eta R^{3}}=\frac{k_{B}T}{6\eta V}$$

where *R* and *V* are the radius and the volume of the rotating sphere, respectively, *η* the viscosity of the solvent, *T* is the temperature and $k_{B}$ the Boltzmann constant. The dependence of the rotational diffusion on the size can be exploited to determine the particle size, e.g., in time-resolved fluorescence polarization or anisotropy experiments. These experimental approaches make use of the fact that the absorption of electromagnetic radiation occurs due to interactions between the electric vector of the incoming light and the dipole moment of the fluorophore. Excitation with linearly polarized laser light thus generates a population of excited fluorophores with a specific orientation of dipole moments, i.e., an anisotropic or polarized distribution, within an isotropic distribution of fluorophores. 

Rotational diffusion of the excited fluorophores, occurring before the emission of a photon at time *t*, leads to a certain amount of depolarization. By measuring the intensity of the emission parallel ($I_{∥}\left(t\right)$) and perpendicular (*I*_⊥_(*t*)) to the polarization of the excitation light the remaining degree of polarization at time *t* can be determined. This can either be expressed as the time-resolved fluorescence polarization *P*(*t*) (Equation (4)):

(4) $$P\left(t\right)=\;\frac{I_{∥}\left(t\right)-I_{⊥}\left(t\right)}{I_{∥}\left(t\right)+I_{⊥}\left(t\right)}$$

or as the time-resolved fluorescence anisotropy *r*(*t*) (Equation (5)):

(5) $$r\left(t\right)=\;\frac{I_{∥}\left(t\right)-I_{⊥}\left(t\right)}{I_{∥}\left(t\right)+2I_{⊥}\left(t\right)}$$

### 2.4. Measurement of Fluorescence Lifetime—Spectroscopy

The fluorescence lifetime of samples in solution can be determined using a time-correlated single photon counting (TCSPC) setup [53]. TCSPC describes a detection principle that allows to determine the time after excitation at which a photon was emitted. In TCSPC measurements the sample is excited with a short, periodic light pulse, e.g., from a picosecond diode laser, white light laser, or Titanium-Sapphire laser with repetition rates in the MHz range. Single emitted photons are then detected. By combining information on the exact time points of the photon detection and the excitation event of that emitted photon, the time after excitation at which the photon was emitted can be obtained. All detected photons then contribute to the histogram of the photon emission times. To accurately reproduce the fluorescence decay curves, sufficient numbers of individual photon recordings are required and hence the process of sample excitation and single photon detection has to be performed a large number of times. To prevent artefacts in the measured fluorescence decay curves the likelihood of having multiple emitted photons per excitation pulse needs to be very small. Therefore, the ratio of photon counts to excitation pulses has to be kept sufficiently low, varying from 1:100 to 1:200 [49,53,54].

### 2.5. Measurement of Fluorescence Lifetime—Microscopy

The principles of measuring fluorescence decay curves with a TCSPC detection system can also be applied to measure the fluorescence decay curves in a spatially resolved fashion, i.e., for each image pixel of a microscope image. Therefore, the excitation light from a pulsed laser source is coupled to a confocal raster scanning microscope [12,15] and single emitted photons are registered by the detector (e.g., single photon avalanche photodiodes, or hybrid detectors [7,12]). 

A confocal raster scanning microscope does not illuminate the whole sample at once, but only a small portion of the sample, the so called confocal volume. A point illumination source, e.g., a laser, is used as excitation light source. The confocal excitation volume is generated by the introduction of a pinhole into the light path. The pinhole is in a conjugated focal plane to the illumination plane within the sample. The main advantage is the suppression of out-of-focus light by the pinhole, even allowing for the acquisition of 3D-images, by scanning the illumination beam both laterally and in the z-plane. To gather information on the whole sample, the confocal volume is moved across the sample, i.e., scanned, and a measurement recorded for each spot. This is usually achieved by a pair of mirrors that can scan the illumination beam across the sample. The whole image can then be reconstructed from the sequential, individual measurements. In essence, this type of confocal TCPSC- based FLIM adds the fluorescence lifetime information to a classical confocal laser scanning microscopy experiment. Two examples of this type of measurement applied to tissue sections will be presented in this review.

Other fluorescence lifetime detection schemes include phase modulation techniques, i.e., using a sinusoidally modulated continuous wave excitation beam, in combination with a wide-field microscope, and wide-field time-gated fluorometry, which amongst others is well suited for phosphorescent probes with lifetimes in the microseconds range [8]. An advantage of FLIM wide-field microscope techniques is the fast acquisition time, thereby, e.g., reducing light damage in the measurements of living cells. 

Multiphoton FLIM measurements allow for non-invasive imaging because of deeper tissue penetration due to the excitation in the near-infrared wavelength region. Similarly, endoscopic FLIM approaches were successfully applied. This allows the imaging, e.g., of human skin physiology and percutaneous drug penetration, as well as clinical applications [55,56,57,58].

## 3. Molecular Properties of Dendritic Nanoparticles

The molecular basis of nanoparticle interactions with cells and tissue, which is key to the targeted delivery of drugs, can only be understood with precise knowledge of the molecular properties of the nanoparticle. Important properties include the size and conformational flexibility of the nanoparticle but also the loading, distribution, and release of drug molecules.

### 3.1. Drug Binding and Release from the Nanoparticle (via Time-Resolved Anisotropy)

For biomedical applications it is of great importance to determine if reporter groups have been bound to the nanoparticle, or if drug molecules have been successfully loaded into the nanoparticle architecture, and also to study if and when these drug molecules are released. As time-resolved fluorescence anisotropy measurements reflect on the conformational freedom of loaded or bound fluorescent molecules, binding of dye conjugates, as well as loading and release of fluorescent drugs and drug mimetics can be investigated. 

The anisotropy decay is commonly described by an exponential decay model function (Equation (6)), yielding a decay time constant, the so-called rotational correlation time *ϕ*:

(6) $$r\left(t\right)=r_{0}\exp\left(-\frac{t}{\phi }\right)$$

where $r_{0}$ is the initial anisotropy at *t* = 0. While a freely rotating small molecule will usually have a fluorescence anisotropy that decays to zero on the subnanosecond timescale with only a single rotational correlation time, a fluorescent dye bound to a larger macromolecule, e.g., a protein or a nanocarrier, will usually display a longer, more complex decay behavior of the fluorescence anisotropy *r*(*t*), described by a multiexponential decay model (Equation (7)):

(7) $$r\left(t\right)=r_{0}\sum \beta_{i}\exp\left(-\frac{t}{\phi_{i}}\right)$$

with $\beta_{i}$ as the amplitude of the *i*^th^ decay component $\phi_{i}$, and $r_{0}$ the initial anisotropy. The extent of rotational diffusion of the dye is limited by the surrounding constituents of the macromolecule, reducing the dye’s motional freedom. Further contributions to the rotational diffusion behavior of the dye can result from the tumbling motion of the whole molecule but also from the segments of the macromolecule to which the fluorescent dye is attached. Therefore, the rotational correlation times $\phi_{i}$ are a measure of different mobilities (e.g., dye, segment, macromolecule), while the amplitudes $\beta_{i}$ of the decay components reflect on the conformational space for the corresponding motion $\phi_{i}$. A common model to describe this restricted rotational diffusion is the “wobbling-in-a-cone” model [59,60]. The time-resolved fluorescence anisotropy of a fluorescent drug or drug mimetic successfully incorporated into the nanoparticle architecture is hence expected to display a multiexponential decay behavior. 

**Figure 4:**
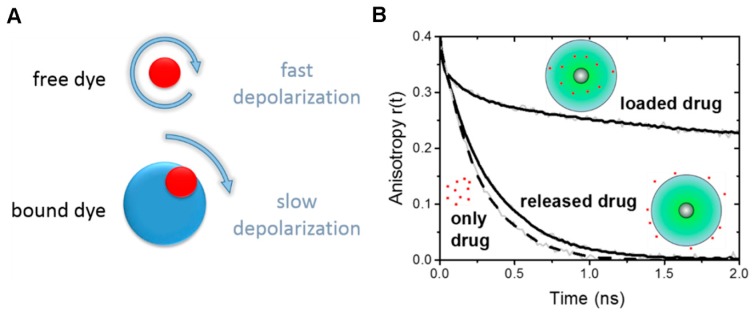
Sensitivity of time-resolved fluorescence anisotropy curves *r*(*t*) for the conjugation state between nanocarrier and fluorescent drug (mimetic). (**A**) Schematic diagram depicting the depolarization rates of free and nanocarrier bound fluorescent dye molecules; (**B**) Example of time-resolved fluorescence anisotropy curves *r*(*t*) of NR, free and loaded onto CMS nanocarriers (CMS/NR). Loaded drug: CMS/NR dissolved in an aqueous solution; released drug: CMS/NR in DMSO; only drug: NR in DMSO. See [61] for more details on the TCSPC based measurements .

Time-resolved fluorescence anisotropy measurements were used to study the model drug mimetic Nile Red loaded into CMS nanocarriers (CMS/NR) [61]. NR was chosen as it hardly fluoresces in aqueous solution. Hence, all observable fluorescence is from NR loaded into CMS when CMS/NR is dissolved in an aqueous solution. Loading of NR into hydrophobic parts of CMS nanocarriers was confirmed by the multiexponential decay behavior of the fluorescence anisotropy for CMS/NR in aqueous solution (Figure 4B, loaded drug). Further, when measuring the time-resolved fluorescence anisotropy for CMS/NR in an apolar solvent (DMSO) the observed anisotropy decay (Figure 4B, released drug) was very similar to NR free in solution (Figure 4B, only drug), indicating nearly free rotation of NR and hence release of NR into solvent.

### 3.2. Nanoparticle Size—Unimer (via Time-Resolved Anisotropy)

Nanoparticles are inherently characterized by their small size. Size determination is a routine procedure for the characterization of nanoparticles. Furthermore, the size of a nanoparticle can have direct implications for its intended use, as size is one of the factors governing the nano-bio interface. Methods to determine the size of nanoparticles include, e.g., electron microscopy, and dynamic light scattering. Time-resolved fluorescence anisotropy measurements also offer the possibility to determine the size of a nanoparticle containing an incorporated fluorescent drug (mimetic) or an attached fluorescent reporter group. The advantage of using time-resolved fluorescence spectroscopy is that the size determination can be performed directly in solution with small sample volumes (e.g., 50 µL are sufficient when using 3 mm × 3 mm quartz cuvettes) and low sample concentrations (in the range of 1–5 µM relating to the fluorophore; the required nanoparticle concentration depends on the respective labeling stoichiometry). 

**Figure 5:**
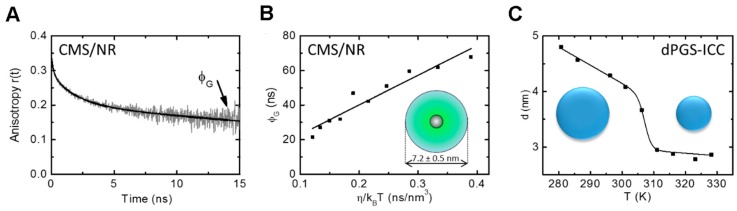
Determining the size of a nanoparticle from fluorescence anisotropy decay curves as measured by TCSPC. (**A**) Exemplary anisotropy decay for CMS/NR. The slowest rotational correlation time $\phi_{G}$ reflecting the tumbling of the whole nanocarrier is indicated; (**B**) Plot of $\phi_{G}$ obtained for CMS/NR against $\eta /k_{B}T$. The volume of the rotating molecule, here CMS, can be obtained from the slope of a linear fit to the data and hence the diameter and radius can be calculated; (**C**) Diameters of dPGS-ICC calculated directly from $\phi_{G}$ as a function of temperature. A shrinkage in size can clearly be seen. (**A**,**B**, adapted with permission from Boreham et al. [61]. © 2014 The American Chemical Society; C, adapted from Boreham et al. [15] by permission of John Wiley and Sons © 2014).

The time-resolved fluorescence anisotropy curve of a nanoparticle with loaded or covalently attached fluorescent reporter groups usually has a multiexponential decay behavior that allows for the assignment of the different main decay components to different modes of rotational motion. For example, the slowest rotational correlation time $\phi_{G}$ (Figure 5A) of the anisotropy decay curve usually describes the rotational diffusion of the whole system and therefore contains information on the size of the particle. The Stokes-Einstein equation (Equation (3)) relates the rotational diffusion coefficient $D_{\mathrm{rot}}$ with the radius *r* of the rotating sphere. Hence, the rotational diffusion depends on the size and shape of the molecule as well as on the viscosity of the solvent. The rotational correlation time of the whole system $\phi_{G}$ is related to the rotational diffusion coefficient *D*_rot_ (Equation (8)) by:

(8) $$\phi_{G}={\left(6D_{\mathrm{rot}}\right)}^{-1}$$

and thus the apparent volume *V* of the rotating sphere can be determined from the measured slowest rotational correlation time $\phi_{G}$ (Equation (9)) by:

(9) $$\phi_{G}=\frac{\eta V}{k_{B}T}$$

Conducting fluorescence anisotropy measurements at different temperatures allows to plot $\phi_{G}$ as a function of $\frac{\eta }{k_{B}T}$ (Figure 5B). The volume, and hence the radius, can then be obtained from a linear fit to the data. For a single temperature the radius can also be directly calculated from the rotational correlation time $\phi_{G}$ (Equation (10)):

(10) $$\frac{k_{B}T\phi_{G}}{\eta }=V=\frac{4}{3}\pi r^{3}$$

Using the information from the slowest rotational correlation time $\phi_{G}$ the size of two dendritic polyglycerol-based nanoparticles [15,61] was determined. The size of CMS nanocarrier unimers was calculated to 7.2 ± 0.5 nm from the fluorescence anisotropy decay data of loaded NR using the slope of the slowest rotational correlation time $\phi_{G}$ plotted as a function of $\eta /k_{B}T$ (Figure 5B) [61]. Application to a dendritic polyglycerol sulfate (dPGS) with a covalently bound indocarbocyanine dye (ICC) revealed a shrinking of the polymer structure when increasing the temperature (Figure 5C) [15].

### 3.3. Drug Partitioning in Nanocarrier (via Fluorescence Lifetime)

The drug partitioning within the nanocarrier architecture can have important consequences for the drug retention and release behavior. Drugs located too close to the surface may be prone to early expulsion from the nanocarrier while drug molecules located deep within the nanocarrier architecture are better protected from expulsion. However, drugs located away from the surface will also require longer release times at the desired release site. Therefore, an understanding of the drug localization within the nanocarrier architecture is necessary for optimized drug retention and release. One possible avenue to investigate the distribution of drug molecules in the nanocarrier architectures is to use a readout method with a high environmental sensitivity. 

The fluorescence lifetime is very sensitive to a fluorophore’s environment. This is mainly due to the strong influence of the environment on the non-radiative decay rate. For example, polar environments tend to reduce the fluorescence lifetime as the non-radiative decay rate is increased due to a higher efficiency of energy transfer to larger dipole moments of the polar environment. Further, some fluorophores exhibit different fluorescence lifetimes depending on their conformation. Here, environmental constraints favoring a certain conformation, such as steric hindrance, can be deduced from the measured fluorescence lifetime [12,15,62].

The fluorescence lifetime *τ* is obtained from an exponential fit to the recorded fluorescence decay curve *I*(*t*) (Equation (11)):

(11) $$I\left(t\right)=I_{0}\exp\left(-\frac{t}{\tau }\right)$$

with $I_{0}$ the intensity at time *t* = 0. However, also more complex fluorescence decay curves can occur, e.g., due to the presence of more than one fluorophore type, the existence of different populations of the same fluorophore or even just because of the molecular structure of the fluorophore itself. These fluorescence decay curves can be approximated by a sum of exponentials according to Equation (12):

(12) $$I\left(t\right)=I_{0}\sum \alpha_{i}\exp\left(-\frac{t}{\tau_{i}}\right)$$

where $\alpha_{i}$ is the respective relative amplitude of the *i*^th^ lifetime component $\tau_{i}$. Due to the high environmental sensitivity of the fluorescence lifetime of a fluorophore, analysis of the fluorescence decay behavior can be used, for example, to determine the distribution of fluorescent drug mimetics in nanocarrier architectures. Nile Red is often used as a hydrophobic drug mimetic [34]. Naturally one would assume that a hydrophobic drug should be located in the hydrophobic parts of the nanocarrier. The following example describes how fluorescence measurements provide information on the rather complex distribution of the fluorescent drug mimetic within the dendritic nanocarrier.

**Figure 6:**
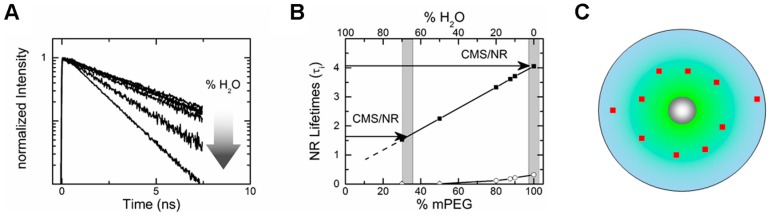
Determining the drug partitioning in CMS nanocarriers from the fluorescence lifetime as measured by TCSPC. (**A**) Fluorescence decay curves of the fluorescent drug mimetic NR in different mixtures of water and mPEG, the outer shell component of CMS. The fluorescence decay clearly depends on the water content of the mPEG solution; (**B**) The lifetimes of NR plotted as a function of mPEG/water mixture. The fluorescence lifetimes found in CMS/NR are a combination of the fluorescence lifetimes of NR in a 30% mPEG and 100% mPEG solution. This indicates that CMS/NR partitions within the outer shell of CMS nanocarriers; (**C**) Schematic representation of the partitioning of the drug within the CMS nanocarrier. The number of incorporated dye molecules is not drawn to scale but to visualize the distribution (adapted with permission from Boreham et al. [61], © 2014 The American Chemical Society).

Based on steady-state fluorescence measurements Fleige et al. [34] had been able to show that NR resides within the outer shell of CMS nanocarrier. However, time-resolved fluorescence measurements (fluorescence lifetime measurements) [61] allowed to determine that NR actually partitions into different regions of the outer shell of CMS nanocarriers (Figure 6). In order to determine the NR partitioning within the outer shell, the fluorescence lifetimes of NR within the CMS nanotransporter were compared to the fluorescence lifetimes obtained for NR in different mPEG/water mixtures (Figure 6A), mimicking the outer shell of the CMS nanocarrier. The results showed that the fluorescence decay of NR within the CMS nanocarrier contains fluorescence lifetime components found both for NR molecules located in a water devoid mPEG mixture and in a mPEG/water mixture with a water content of 70%, suggesting a distribution of NR within the outer mPEG shell of the CMS nanotransporter (Figure 6B). Furthermore, the distribution of the NR molecules in these two regions could be determined from the amplitudes of the respective lifetimes, with 78% of NR molecules residing away from the surface at 20 °C. In addition, by measuring the fluorescence decay of NR in CMS nanocarriers dissolved in other solvents it was shown that the polarity of the solvent affects the drug distribution in the outer shell. Similarly, binding of biomolecules to dendritic nanocarriers (e.g., protein corona) may be observed by the changes of the fluorescence lifetime of the nanocarrier bound fluorophore [62].

### 3.4. Conformational Flexibility of Nanocarriers (via Time-Resolved Anisotropy)

To fully understand the molecular interaction of nanocarrier branches with cargo it is important to gain insights into the molecular dynamics and conformational flexibility of the nanocarrier itself. The local rigidity or flexibility, and the resulting conformational flexibility of the nanoparticle architecture can have direct consequences for guest molecules and their localization within or their release from the nanocarrier. Further, segmental dynamics of polymer branches can be regarded as an important factor for interactions with constituents of tissues and cells. Both the segmental dynamics and the steric restriction of motions can be obtained from time-resolved fluorescence anisotropy measurements. The fluorescent reporter group can either be covalently linked to or loaded onto the nanoparticle. Depending on the nanocarrier architecture and site of covalent attachment, the covalently bound fluorophore and the loaded fluorescent drug (mimetic) may report on different regions of the nanocarrier structure. 

In a recent study [15], the segmental dynamics of ICC attached to the surface of dPGS was investigated. The segmental dynamics, as inferred from the bound ICC dye, was shown to be temperature independent, in contrast to the size of dPGS. Further, the conformational space of the bound ICC dye also did not depend on the temperature. From the data on the conformational flexibility of dPGS-ICC, it was concluded that the temperature-dependent size change originates from the core of dPGS. 

**Figure 7:**
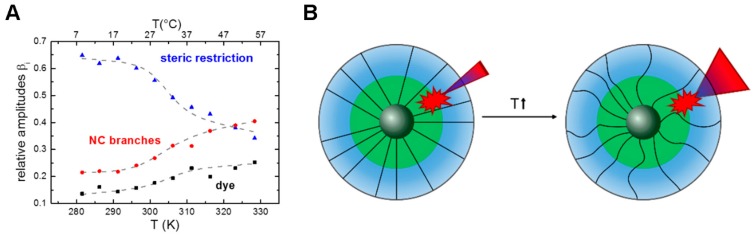
Relative amplitudes of the fluorescence anisotropy decay reflect on the conformational flexibility of nanoparticles. This example shows the temperature dependent changes in conformational flexibility of the CMS nanocarrier. (**A**) The relative amplitudes β_i_ of the fluorescence anisotropy decay CMS/NR reflect on the motional freedom of the dye, the conformational flexibility of the nanocarrier branches and the overall steric restriction; (**B**) Schematic representation of the increase of conformational space (indicated by the cone size) of NR as well as the increased flexibility of NC branches with increasing temperature. (**A**, adapted with permission from Boreham et al. [61], © 2014 The American Chemical Society).

Pronounced changes in the conformational flexibility of a nanoparticle were observed for CMS nanocarriers [61]. Here, the anisotropy decay component $\phi_{2}$ (Equation (7)) attributed to the dynamics of the nanocarrier branches (red data points in Figure 7A) showed an increase in the relative amplitudes with increasing temperature, indicating that the nanocarrier branches require more conformational space at higher temperatures (Figure 7B). This result was interpreted as increased flexibility of the nanocarrier branches at higher temperatures (Figure 7B) [61].

### 3.5. Conformational Transitions Affecting Drug Partitioning (Temperature Dependent Anisotropy, Temperature Dependent Lifetime, Two-State Transitions)

Changes in the molecular structure of the nanocarrier can of course have direct consequences on the distribution of guest molecules within the nanocarrier. Such changes may be induced by altering the environment, e.g., temperature, for CMS nanocarriers. The use of both time-resolved fluorescence lifetime and anisotropy measurements allows to simultaneously monitor the changes in the distribution of a loaded fluorescent drug mimetic and the conformational flexibility of the nanocarrier itself. A direct correlation between conformational changes and drug partitioning can then be obtained. Depending on the transition behavior, appropriate models can be selected that can further characterize the observed transition with regard to thermodynamic parameters, e.g., the transition temperature.

**Figure 8:**
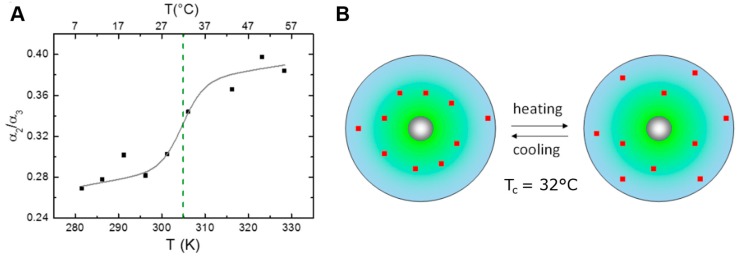
Drug partitioning in CMS/NR as a function of temperature. (**A**) The ratio of the relative amplitudes α_2_ and α_3_ reflects on the ratio of NR close to the surface and near the hydrophobic inner shell of CMS. With increasing temperature NR partitioning is shifted in favor of surface vicinity. The dashed green line indicates the transition temperature determined from a fit of the data with a two-state transition model; (**B**) Schematic representation of the redistribution of NR within the different shells (blue—hydrophilic, green—hydrophobic, grey—PG-core) of CMS occurring with temperature. The number of incorporated dye molecules (approximately one per CMS structure) is not drawn to scale but to visualize the distribution (adapted with permission from Boreham et al. [61], © 2014 The American Chemical Society).

In the case of NR loaded CMS nanocarriers it was shown from temperature dependent fluorescence anisotropy measurements that temperature induced conformational transitions occur [61] (Figure 7B). At the same time, the ratio of the fluorescence lifetimes reflecting on the NR partitioning also exhibited a temperature dependent behavior, with more NR molecules near the CMS/water interface at higher temperatures. Both transitions were approximated with a two-state transition model [15,61,63,64] (Figure 7 and Figure 8), revealing that the phase transition in the CMS nanocarrier branch dynamics leads to a shift in the distribution of the encapsulated hydrophobic cargo molecule from the CMS interior toward the outer shell/solvent interface at higher temperatures (Figure 8B).

## 4. From Molecular to Cellular/Tissue Insight

Understanding the behavior of a nanoparticle in vivo is of fundamental importance for the application in nanomedicine. Biodistribution and metabolic clearance are both directly related to cytotoxicity and biocompatibility of nanoparticles and biomolecules, while a deeper understanding of the principles governing the behavior at interfaces as well as the transport of substances across barriers is required for the rational design of new drug delivery vehicles. In this context biomolecular interactions of the respective nanoparticle are of utmost importance. In the case of dPGS, the dendritic nanoparticle was shown to be anti-inflammatory active through the binding of dPGS to selectins [26,30], i.e., the interactions of dPGS in blood serum play a role. Using the unique fluorescence lifetime of ICC bound to dPGS, time-resolved fluorescence lifetime experiments provided evidence that dPGS recognizes L-selectin despite the presence of other competing proteins in blood plasma [62].

Fluorescence lifetime imaging microscopy (FLIM) can be used to spatially resolve fluorescent molecules based on their fluorescence lifetime, gaining additional contrast compared to conventional fluorescence microscopy. Furthermore, FLIM can help visualizing interaction processes with various biomolecules, based on the changes in fluorescence lifetimes upon interaction. However, the poor signal-to-noise ratios and high fluorescent backgrounds, e.g., common in FLIM imaging of skin and tissue samples, are a challenge for FLIM data analysis [15].

### 4.1. Distinguishing Between Nanoparticles and Autofluorescence Using the Unique Fluorescence Lifetime Signature 

**Figure 9:**
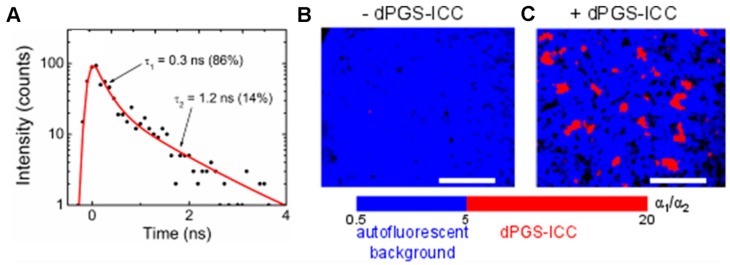
Examples of distinguishing of fluorescently labeled nanoparticles from tissue autofluorescence using unique fluorescence lifetime signatures as measured by TCSPC in a FLIM setup. (**A**) Unique fluorescence lifetime signature of dPGS-ICC. The lifetimes and relative amplitudes are given; (**B,C**) Ratiometric FLIM analysis detecting dPGS-ICC in liver tissue. The specific lifetime amplitude ratio for dPGS-ICC (red) was only observed in tissue samples containing dPGS-ICC (**C**) and not in the control sample (**B**). Scale bar: 50 μm (Reprinted (adapted) from Boreham et al. [12] with permission of The American Chemical Society, © 2011).

The unique fluorescence lifetime signature of fluorescently labeled nanoparticles can be used to separate them from the background autofluorescence. Recently a number of publications by our group have reported on successful detection of nanoparticles in tissue samples based on unique fluorescence lifetime signatures [11,12,13,14,16,61]. A ratiometric FLIM approach [12] used the amplitude ratio of the two fluorescence decay components in the fluorescence lifetime signature of the target molecule dPGS-ICC to separate dPGS-ICC fluorescence from liver tissue autofluorescence (Figure 9A). Localization of dPGS-ICC to Kupffer cells in the liver could thus be shown [12]. However, when attempting to detect dPGS-ICC specifically within cell membranes based on the unique fluorescence lifetime signature determined in a vesicular lipid membrane system, it was found that the ratiometric FLIM approach is limited to target molecules with no more than two lifetime components [15]. Further studies of our group made use of a multivariate pattern recognition method to partition the fluorescence decay curves into classes. Application of this new FLIM data analysis method enabled the identification of penetration of ICC labeled CMS into skin samples [11] and the localization of FITC labeled silica nanoparticles in mouse tissue sections [13,14].

### 4.2. Unique Fluorescence Lifetime Signature as a Tool to Detect Environmental Effects

**Figure 10:**
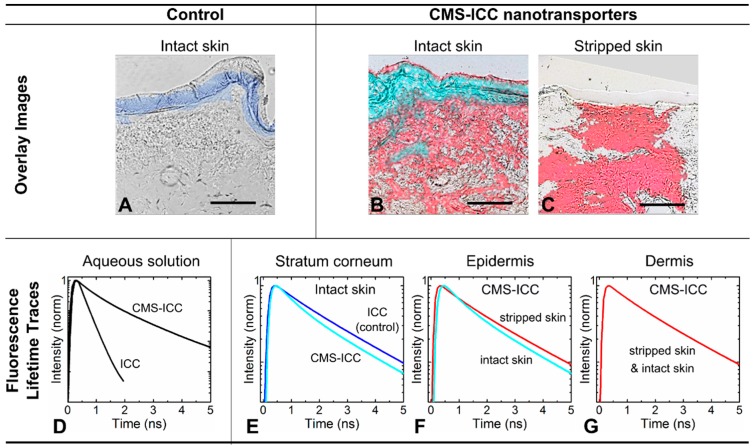
Fluorescence lifetime microscopy (FLIM) of CMS–ICC nanotransporters. CMS–ICC nanotransporters were applied onto normal and stripped human skin for 24 h. Representative overlay images (FLIM and bright field) of the same area are shown: (**A**), As a control, an aqueous ICC solution was applied to normal skin. CMS-ICC nanotransporters were applied to (**B**), normal skin and (**C**), stripped skin. The false color coding is based on the unique fluorescence lifetimes of CMS–ICC nanotransporters (cyan, red) or ICC (blue) in the different skin layers. (**D**), the fluorescence lifetime traces (as measured by TCSPC) of CMS–ICC nanotransporters and ICC in aqueous solution are shown. (**E**–**G**), The fluorescence lifetime traces of CMS–ICC nanotransporters and ICC are shown in the same colors as used in the corresponding overlay images (**A**–**C**). All lifetime traces are normalized to 1. Scale bar: 100 μm (reprinted from Alnasif et al. [11] with permission from Elsevier, © 2014).

As the fluorescence lifetime is a very sensitive tool for environmental changes, FLIM can also be employed to detect environmental effects in tissue samples. If such environmental effects are restricted to certain characteristic regions of a tissue sample, these may hint to specific interactions with proteins or lipids. Hence, FLIM analysis methods capable of resolving these differences in the fluorescence lifetime signatures are needed. Examples are the penetration of CMS-ICC (Figure 10) [11] or hyaluronic acid [16] into skin samples with a normal or a deficient barrier. While CMS-ICC [11] in barrier deficient skin and deeper layers of normal barrier skin all showed the same fluorescence lifetime signature, a further CMS-ICC specific fluorescence lifetime signature was observed exclusively in the upper skin layer, the stratum corneum, of normal barrier skin. These observed differences between the fluorescence lifetime curves in stratum corneum and deeper skin layers indicate striking interactions of the carrier with the lipids or proteins of the stratum corneum.

## 5. Conclusions

In conclusion, time-resolved fluorescence techniques are powerful tools at the interface between chemical/material science and biological application for the new task presented by the emerging field of nanomedicine. The application of time-resolved fluorescence spectroscopy and fluorescence lifetime imaging (FLIM) for the analysis of nanoparticles allows to gain new insights, from the molecular up to the tissue level. On a molecular level, the sensitivity of the fluorescence lifetime to changes in a fluorophores environment facilitates analysis of the drug partitioning in nanocarriers while measurements of the time-resolved fluorescence anisotropy can report on the successful binding or loading of molecules to nanoparticles, on drug release, and also on the size and conformational flexibility of nanoparticles. Environment specific unique fluorescence decay curves [62] of fluorescent reporter groups attached to or loaded onto nanoparticles allow to distinguish reporter group fluorescence from autofluorescence in cell and tissue samples [11,12,13,14,15,16]. This enables, for example, investigating the biodistribution in tissue, skin penetration, and environmental effects in cells and tissue. Moreover, when using the appropriate experimental approaches it is even possible to quantify particle amounts and detect close interactions of labeled molecules.

## Abbreviations

The following abbreviations are used in this manuscript:

CMS: Core-multi-shell
CMS-ICC: Indocarbocyanine bound to core-multi-shell nanocarrier
dPGS: Dendritic polyglycerol sulfate
dPGS-ICC: Indocarbocyanine bound to dendritic polyglycerol sulfate
FLIM: Fluorescence lifetime imaging microscopy
FRET: Förster resonance energy tranfer
ICC: Indocarbocyanine
TCSPC: Time-correlated single photon counting
